# Researching health in diverse neighbourhoods: critical reflection on the use of a community research model in Uppsala, Sweden

**DOI:** 10.1186/s13104-018-3717-7

**Published:** 2018-08-25

**Authors:** Sarah Hamed, Sonja Klingberg, Amina Jama Mahmud, Hannah Bradby

**Affiliations:** 1 Department of Sociology, Uppsala University, Uppsala, Sweden; 2 MRC Epidemiology Unit, University of Cambridge, Cambridge, UK; 3 Independent Researcher, Karlskrona, Sweden

**Keywords:** Sweden, Healthcare research, Community research

## Abstract

**Objective:**

A community research model developed in the United Kingdom was adopted in a multi-country study of health in diverse neighbourhoods in European cities, including Sweden. This paper describes the challenges and opportunities of using this model in Sweden.

**Results:**

In Sweden, five community researchers were recruited and trained to facilitate access to diverse groups in the two study neighbourhoods, including ethnic, religious, and linguistic minorities. Community researchers recruited participants from the neighbourhoods, and assisted during semi-structured interviews. Their local networks, and knowledge were invaluable for contextualising the study and finding participants. Various factors made it difficult to fully apply the model in Sweden. The study took place when an unprecedented number of asylum-seekers were arriving in Sweden, and potential collaborators’ time was taken up in meeting their needs. Employment on short-term, temporary contracts is difficult since Swedish Universities are public authorities. Strong expectations of stable full-time employment, make flexible part-time work undesirable. The community research model was only partly successful in embedding the research project as a collaboration between community members and the University. While there was interest and some involvement from neighbourhood residents, the research remained University-led with a limited sense of community ownership.

## Introduction

This paper explores the use of a community research model adopted in Sweden as part of a multi-country healthcare project [1]. The model defines community research as “the practice of engaging community members as co-researchers to research issues within their own communities with a view of accessing community specific issues”, to offer community members ownership of the research agenda, and facilitate access to specific issues or groups [2]. Different partners with different skills, understandings and experiences can address complex problems while improving the quality, validity and rigour of research and ensuring its continuity and sustainability [3]. Community research models may be particularly useful in diverse and deprived settings occupied by so-called “hard to reach” communities. Community researchers who are multilingual and have access to and knowledge of their own communities may improve recruitment and retention efforts [4].

However, community research models potentially involve complex power dynamics and discordant expectations [5], and it is in this light that we discuss the model for research practice and findings of our study in Sweden. The community research model was part of the UPWEB^1^ project exploring access to healthcare in diverse neighbourhoods. Community research is seldom used in Sweden: we have not found any published articles critically discussing its use here. This paper discusses challenges and lessons that can inform future applications of community research models, particularly in increasingly diverse research settings.

## Main text

### Materials and methods

The UPWEB project investigated access to healthcare in eight diverse neighbourhoods in four European countries (Sweden, Germany, Portugal, UK). The project aim was to understand how residents of superdiverse areas access healthcare, with specific aims to:

- Examine residents’ experiences of accessing and communicating with healthcare providers and the approaches residents take to optimize their access to healthcare.
- Investigate the factors influencing people’s access to, and experiences of, healthcare including local and national welfare states, health and migration regimes.
- Explore the ways in which different types of providers identify need and investigate the roles they adopt, and challenges and opportunities they face.
- Use the experiential knowledge of providers and residents to develop new models of provision and test the applicability of these models to a wider population.

The two Swedish neighbourhoods were in Uppsala—Sweden’s fourth largest city—both highly diverse, comprising a mixture of long-term and recent residents of Swedish origins, and different migrant groups, both recent arrivals and those who had come to Sweden decades earlier. Diversified diversity was apparent, not only in terms of ethnicity, but also in terms of socioeconomic status, immigrant status, participation in the labour market, age, gender, and religion [6]. Both neighbourhoods in Uppsala were deprived with higher levels of poverty and annual sick leave days than the city average [7]. Table 1 below shows characteristics of these neighbourhoods in comparison to Uppsala municipality [8].

**Table 1:** Neighbourhood characteristics

	Uppsala	Neighbourhood 1 (Gottsunda)	Neighbourhood 2 (Sävja)
Location	67 km north of Stockholm	7 km south west of city centre	6 km south east of the city centre
Population in 2016	214 559	10 198	6519
People with a foreign background	52,649 (24%)	5568 (54.5%)	2188 (29.7%)
Total average annual earnings^a^ in 2015^b^, thousand Swedish crowns	327.7	226	290
Unemployment rate in 2016 (16–64 years)	5.5%	8.6%	5.3%
Sick days^c^ in 2015	22.3	35.6	29.6

^a^Average earnings is the sum of income from employment and business. Consists of the total current taxable income from employment, entrepreneurship, pension, sickness benefit and other taxable transfers. Does not include income from capital

^b^No statistics exist for 2016

^c^Sick days is the Swedish Social Insurance Agency’s measure of the number of days of absence from work compensated by sickness benefit during a 12-month period

The project used mixed methods including ethnographic observations, in-depth qualitative interviews (with healthcare users and providers in the neighbourhoods who were not financially compensated) and a quantitative survey. Community researchers were recruited at the beginning of the project to carry out both ethnographic observations and in-depth interviews alongside the academic research team.

The project’s community research model was developed in the UK by Goodson and Phillimore [2] and adopted in the other three countries. The aim was to recruit local community members from key minorities, train them in qualitative research methods, and support them in engaging their own communities in research. Community researchers could introduce the research project to their networks, map the locality, recruit interviewees, translate interviews and, in some cases, conduct and transcribe interviews. The model had originally been developed for researching “hard to reach” communities, such as refugees, in the UK.

#### Recruitment and training of community researchers in Sweden

The position of community researcher was advertised locally in Uppsala. Established contacts from the study neighbourhoods were encouraged to apply. The vacancy was also advertised in the neighbourhoods’ cultural centres and Uppsala University’s Department of Public Health.

Five community researchers were recruited speaking various languages other than Swedish, including English, Finnish, Somali, Swahili and Arabic. Community researchers were hired on a short-term temporary hourly basis. Two days of training in qualitative research methods, including interviewing, ethics and transcription, and an introduction to the project were conducted by the main project researchers in Sweden. Two of the community researchers did not reside in the study neighbourhoods but had language and research skills that were useful to the project. See Table 2 for further details of the community researchers.

**Table 2:** Characteristics of community researchers

Community researcher	Age group	Educational level	Residency at the time of the study	Languages spoken	Other employment at the time of the study
1	20–30	Master degree in public health	Uppsala outside of the neighbourhoods	Swedish, English, Finnish	None
2	20–30	Bachelor degree	Uppsala outside of the neighbourhoods	Swedish, English, Somali	None
3	20–30	Secondary school	Neighbourhood 2	Swedish, Arabic	Employed at a leisure centre
4	20–30	Master degree in public health	Neighbourhood 1	Swedish, English, Arabic	None
5	40–50	Bachelor degree	Neighbourhood 1	Swedish, English, Swahili, Somali	Manager at a citizen education organisation

Overall, the community researchers assisted in recruiting and interviewing 35 healthcare users and 15 healthcare providers and Table 3 shows some of their characteristics.

**Table 3:** Healthcare users’ characteristics

Total number of interviewees	35
Gender	
Males	15
Females	20
Marital status	
Married	16
Divorced	4
Widowed	5
Single	4
In a relationship	2
Not known	4
Religion	
Muslim	14
Christian	9
Agnostic	2
Atheist	1
Not relevant	6
No religion	3
Employment	
Employed	17
Retired	6
Stay at home	3
Student	3
On sick leave	2
Unemployed	4
Countries of birth	
Ukraine	1
Chile	2
Indonesia	1
Iran	2
Iraq	2
Sudan	2
Kenya	1
Somalia	4
Inkerinmaa (Finland)	1
Lebanon	1
Palestine	2
Syria	3
Sweden	13
Age in years	
20–30	7
31–41	7
42–52	5
53–63	6
64–74	5
75–85	4
86–96	1
Health concerns	
Uterine Myoma	1
Diabetes	4
Hypertonia	2
Heart failure	2
Other heart problems	3
Mental illness	7
Missing tooth	1
Hand injury	1
Arthritis	1
Epilepsy	1
Hypothyroidism	1
Fibromyalgia	1
Chronic obstructive pulmonary disease	1
Haemorrhoids	1
Rheumatism	2
Lung embolism	1
Asthma	3
Dental caries	1
Impaired kidney function	1
Chronic pain	4
Allergies	1
Eye condition	1
Irritable bowel syndrome	1
Multiple sclerosis	1
Languages spoken	
Swedish	32
Finnish	1
English	20
Greek	1
Italian	1
Indonesian	1
Hindi	1
Chinese	1
Arabic	7
Kurdish	2
German	3
Persian	2
Swahili	2
Tigrigna	2
Amharic	1
Somali	4
Kikuyu	1
Russian	3
Ukrainian	1
Spanish	3
German	2
French	3
Norwegian	1
Dutch	1
Italian	1

Public health and health promotion in Sweden is integral to society and community researchers assisted in recruiting and interviewing 10 women and 5 men healthcare providers from education, youth work, sport and library services as well as primary care, midwifery, pharmacy, elder care and psychiatry. Due to confidentiality issues, further individual identifying information for the healthcare providers is withheld.

## Community research in action

Community researchers recruited study participants from the neighbourhoods, independently conducted or assisted during interviews and carried out ethnographic observations. Their individual involvement and interest in the project varied considerably. For various reasons it was difficult to recruit and retain community researchers in Sweden. The project mainly attracted applications from unemployed graduates. Only one community researcher who already had an interest in research (and a master’s degree in international health) worked with the project until all interviews were conducted and transcribed.

The community researchers’ local networks and knowledge about the neighbourhoods were invaluable for contextualizing the study and identifying and recruiting participants. While community researchers facilitated access to some groups, they were less able to reach and recruit others groups (such as intra-European migrants including Roma) due to language barriers and the limits of their networks. The limited number of community researchers appointed from the start was partly due to the high cost and bureaucratic burden of hiring community researchers in the Swedish context. Moreover, benefits to the individuals who were undertaking community research were not apparent. The community research model assumed certain ideal conditions for its implementation, which were not present and which, together with various contextual factors, contributed to problems in adopting the model in Sweden and are discussed below.

### Ideal conditions

#### What community means

This project sampled by neighbourhood, which in Sweden implies clearly delimited urban areas built around specific facilities such as schools, library, healthcare centre, etc. The research project assumed that people living in such a neighbourhood considered it not just a place of residence, but also identified with the locality with some sense of community.

Defining the community as both a locality and an identity, assumed that people living in the neighbourhood had some sense of community in relation to the geographical location. In both Swedish neighbourhoods, community researchers did describe a shared community identity but they had different perspectives on how this manifested itself. For some it was a positive sense of belonging whereas for others it was more negatively expressed as a feeling of “us against the world”. Both neighbourhoods are very diverse, and to some extent socioeconomically segregated and racialised [9] with residents of certain areas within the neighbourhood wanting to distinguish themselves from the rest of the neighbourhood. A community is not homogenous and researchers identified differently with the community, as residents and non-residents, offering different versions of an “insider” perspective.

#### Community ownership

The community research model [2] assumes that there is both interest in and capacity for participation among community members who can contribute to, and get something out of participating in research [10]. However, in the Swedish case community researchers were not fully part of the research process, for instance not having contributed to the research design and the choice of methods. While empowerment and co-ownership are central aspects of the community research model, we found the possibilities for this were limited. Two active community researchers, who were curious about how research could feed into policy and practice, got involved in the project, but the community research model was only partly successful in embedding the project as a collaboration between community members and the University. The research remained University-led with a top-down approach contributing to a limited sense of community ownership.

### Contextual factors

#### Refugee arrivals

The study took place during 2015–2016 when 163,000 asylum-seekers arrived in Sweden [11]. The need to find housing, access education and other services for these new arrivals, many of whom spoke neither English nor Swedish, put considerable strain on local authorities, community organisations, and cooperative associations (*föreningar*) in the study neighbourhoods. In particular, the arrival of unaccompanied refugee minors during the summer of 2015 [12] kept key contacts in both neighbourhoods fully occupied. These key contacts were the very people with valuable “insider knowledge” who might have otherwise have been employed as community researchers.

#### Employment conditions

Employing several people on short-term contracts is complicated as Swedish Universities are public authorities or state agencies (*myndighet*) with all the bureaucracy that comes with being a state institution [13]. Temporary, part-time work without a contract can be offered on an hourly paid basis^2^ to students and others where the work is assumed not to be their main source of income. There is a strong expectation of stable full-time employment in the Swedish labour market and flexible part-time work is seen as undesirable. Short-term contracts are complicated and expensive to administer, as all state employees are entitled to benefits such as paid leave, and after a period of time temporary contracts are expected to be made permanent [14]. Since the cost of living and the level of personal income tax are high [15], it is difficult to survive with short-term temporary hourly work, as was available for community researchers. As was already mentioned, project-based employment was possible but beyond the bureaucratic challenges, it was not attractive to potential community researchers, who tended to prefer more reliable forms of employment with more remunerative and secure longer-term prospects, even if they had an interest in the research.

### Conclusion

Community research is beneficial in accessing participants and promoting co-ownership of research, especially in deprived areas that might otherwise be excluded from research. Ideally community research should begin with a topic that originates from the community itself [5] which was not the case in this project. The community researchers were not involved in the proposal writing and interview preparation, which limited the possibility of developing co-ownership, and may have contributed to a lack of enthusiasm. Thus, community researchers’ interests were not embedded in the research from its outset and no real shift in the traditional power dynamics of research occurred. This raises the question of how community research can work within an institutional setting [16], especially in a country such as Sweden where universities are bureaucratic public agencies.

Community participation in research is not necessarily a need or demand arising from within a community but may rather reflect academic discourse on the ethics of knowledge production and innovative research methods. Having a sense of shared interests with one’s neighbourhood cannot be assumed, and even where a sense of community exists, equitable participation in research is difficult due to power imbalances within a community.

## Limitations

The community research model can be interrogated in terms of assumptions about community and context-specific factors of implementation. In terms of identifying and recruiting research participants, relying on community researchers’ networks may lead to convenience sampling rather than an agreed sampling strategy. Poor research validity and reliability, have been highlighted as challenges of community research models [5]. While training community researchers and paired interviewing supports validity and reliability, more extensive training and regularly debriefing, especially for those new to research are recommended. Models of community research are specific to culture and place and cannot be simply exported to another context. A context analysis may support applicability and sustainability of community research.

## Abbreviations

UPWEB: understanding the practice and developing the concept of welfare bricolage
UK: United Kingdom

## Footnotes

1 https://www.birmingham.ac.uk/generic/upweb/index.aspx.

2 Hourly rates in 2016 were paid at approximately 13 Euros per hour, plus 12% vacation pay.
